# Musical Experience, Sensorineural Auditory Processing, and Reading Subskills in Adults

**DOI:** 10.3390/brainsci8050077

**Published:** 2018-04-27

**Authors:** Parker Tichko, Erika Skoe

**Affiliations:** 1 Department of Psychological Sciences, Developmental Psychology Division, University of Connecticut, Storrs, CT 06269, USA; parker.tichko@uconn.edu; 2 Department of Speech, Language, and Hearing Sciences, University of Connecticut, Storrs, CT 06269, USA; 3 Connecticut Institute for the Brain and Cognitive Sciences, University of Connecticut, Storrs, CT 06269, USA

**Keywords:** reading development, auditory processing, auditory brainstem response, musical training, phonological awareness, rapid naming

## Abstract

Developmental research suggests that sensorineural auditory processing, reading subskills (e.g., phonological awareness and rapid naming), and musical experience are related during early periods of reading development. Interestingly, recent work suggests that these relations may extend into adulthood, with indices of sensorineural auditory processing relating to global reading ability. However, it is largely unknown whether sensorineural auditory processing relates to specific reading subskills, such as phonological awareness and rapid naming, as well as musical experience in mature readers. To address this question, we recorded electrophysiological responses to a repeating click (auditory stimulus) in a sample of adult readers. We then investigated relations between electrophysiological responses to sound, reading subskills, and musical experience in this same set of adult readers. Analyses suggest that sensorineural auditory processing, reading subskills, and musical experience are related in adulthood, with faster neural conduction times and greater musical experience associated with stronger rapid-naming skills. These results are similar to the developmental findings that suggest reading subskills are related to sensorineural auditory processing and musical experience in children.

## 1. Introduction

The development of literacy hinges on the acquisition of several rudimentary reading subskills, namely phonological awareness (i.e., explicit knowledge about the sound structure of spoken language) and rapid naming (i.e., the ability to rapidly decode written symbols into sound) [1,2,3]. For emerging readers who are simultaneously learning a spoken language, acquiring reading subskills, such as phonological awareness and sound-to-symbol mapping, is thought to be partially mediated by basic auditory-processing mechanisms that track auditory information on multiple timescales in the speech signal, such as rapid acoustic changes (e.g., formant transitions, phonemic information) and prosodic information (e.g., amplitude rise times, vowel pitch contour, syllabic information) [4,5,6,7,8,9,10,11,12]. Consequently, reading theorists have proposed that the development of the auditory system is inextricably linked to the development of reading-related skills, particularly for children acquiring a spoken language [8,12,13,14,15,16,17,18,19,20,21,22].

Importantly, the development of auditory skills underlying literacy (e.g., rapid auditory processing, tracking prosodic dynamics) relies on the maturational and functional properties of the human central auditory system; a network of neural structures that span from the cochlear nucleus in the pontomedullary junction in the brainstem to the primary auditory cortex in the temporal lobe. Indeed, a growing body of work suggests that sensorineural auditory processing in central auditory structures is related to reading ability across the lifespan, beginning in the pre-literate period and continuing into adulthood [17,23,24,25,26,27,28]. For instance, in children with clinically-normal hearing, poorer speech-sound decoding and reading fluency are both associated with weakened sensorineural encoding of speech [23,24,28]. Moreover, children with poor reading skills, have poorer neural differentiation of speech syllables and more variable neural responses to speech sounds, relative to children with good reading skills [17,29]. (Note, however, that this variability in auditory processing may be reduced through short-term auditory training [30].) Recent work also suggests that relationships between reading ability and sensorineural auditory processing persist into later stages of development. For instance, adult readers with more biologically mature subcortical auditory-evoked potentials were reported to have globally better performance across a composite measure of reading subskills [31].

If sensorineural auditory processing plays a significant role in reading development, it is possible that general auditory skills or other forms of auditory training that enhance sensorineural auditory processing might likewise influence reading outcomes. Musical training, for instance, is an auditory-based, sensorimotor activity that children often participate in during the formative years of reading development. Research suggests that musical training sharpens sensorineural processing of sound across the lifespan [15,32,33,34,35]. For instance, children and adults who have undergone musical training early in life often exhibit more robust indices of sensorineural auditory processing [32,35,36]: Adult musicians, compared to adult non-musicians, have faster neural response latencies and a more faithful neural representation of sound [35,36,37]. Moreover, musicians, compared to non-musicians, exhibit enhanced neural differentiation of speech sounds [38,39], less variable neural responses to speech, and better neural tracking of the dynamic properties of complex sounds [36,40,41,42,43]. While auditory-processing enhancements have been mostly investigated with complex sounds, there is some evidence that musical training is also associated with faster neural responses to transient stimuli, such broadband, click stimuli [35]. Similar sensorineural-auditory-processing enhancements have also been observed with adolescents [33] and children undergoing musical training [44]. Given, in part, these findings, several authors have theorized that musical training might bolster shared, underlying sensorineural auditory processing mechanisms important for reading development and speech processing [4,12,18,45,46].

In addition to enhancing sensorineural processing skills, musical training may bootstrap higher level reading subskills, such as phonological awareness and rapid naming [47,48,49,50,51]. For example, given that both spoken language and music are sound-based combinatorial systems, learning to manipulate melodic and rhythmic structures in music may confer benefits to analogous sound structures of spoken language (e.g., phonology) [50]. Similarly, both the sound structure of spoken language and music can be symbolically represented in written form, using orthographic systems or formal music notation. Thus, it is also possible that formal musical training and sight reading may benefit the ability to decode other symbolic representations of sound, such as orthography [47,52].

Consistent with these proposals, developmental research suggests that music skills and reading subskills, such as phonological awareness and rapid naming, are intimately related in childhood [5,25,47,48,49,50,51,53]. For instance, in one study, preschoolers’ rhythm and pitch abilities (e.g., rhythm production, rhythm discrimination, melody discrimination, chord discrimination) were correlated with measures of phonological awareness and letter identification [47], while, in another study, preschoolers’ ability to entrain to a musical beat was found to be associated with phonological-awareness and rapid-naming abilities [54]. In addition to reading subskills, other work has reported relations between musical skills and more general reading abilities [55]. Moreover, in cases of atypical reading development, such as developmental dyslexia, impairments in rhythm, meter, and pitch perception, skills fundamental to music cognition and perception, are commonly reported [56]. Finally, a growing body of experimental work suggests that music- and rhythm-based interventions may bolster reading development in both typical and atypical readers [55,57,58,59,60,61,62,63].

While previous work on sensorineural auditory processing, musical training, and reading development has typically been studied emerging readers, it is unknown whether musical training is also related to sensorineural auditory processing and reading subskills in mature readers. Whilst, to our knowledge, no research has collectively linked sensorineural auditory processing, reading subskills, and musical training in a single sample of adult readers, several studies do suggest a link between measures of auditory processing, musical training, and reading ability in adulthood: for instance, adult musicians are more sensitive to speech rhythms relative to non-musicians [64]. Moreover, adult musicians with dyslexia, compared to non-musicians with dyslexia, have better basic auditory processing (e.g., rise time onset detection; perception of intensity, rhythm, and frequency) and reading subskills (e.g., word and non-word naming) [65,66].

In the current study, we investigated whether a history of musical training related to sensorineural auditory processing and reading subskills in a sample of adult readers. Considering evidence that musical experience and musical aptitude relates to children’s rapid-naming and phonological-awareness skills [47,50,54,67], we specifically hypothesized that earlier musical training, longer musical training, greater proficiency of musical skills, and more recent musical training would be associated with stronger rapid-naming and phonological-awareness abilities in adulthood. Moreover, we predicted that sensorineural auditory processing, as assessed by click-evoked auditory-brainstem responses (ABRs), would relate to both reading subskills and a history of musical training. Here, we measured neural conduction times and the consistency of the sensorineural response to a click sound [17,35]. We also adopted a paradigm to test whether relationships between sensorineural auditory processing and reading subskills were related to the phonemic or syllabic level of temporal speech processing [68,69,70] by presenting auditory stimuli at different presentation rates.

The analysis, which treats musical training as a set of continuous variables, is structured to answer three research questions: (1) Is sensorineural auditory processing related to phonological and rapid-naming skills in adulthood? (2) Do differences in musical training history relate to phonological and rapid-naming skills in adulthood? (3) Do differences in musical training history relate to sensorineural auditory processing? Here, we report results from a single sample of adult readers with varying histories of musical training.

## 2. Materials and Methods

### 2.1. Auditory Brainstem Response (ABR) Protocol

**Figure 1:**
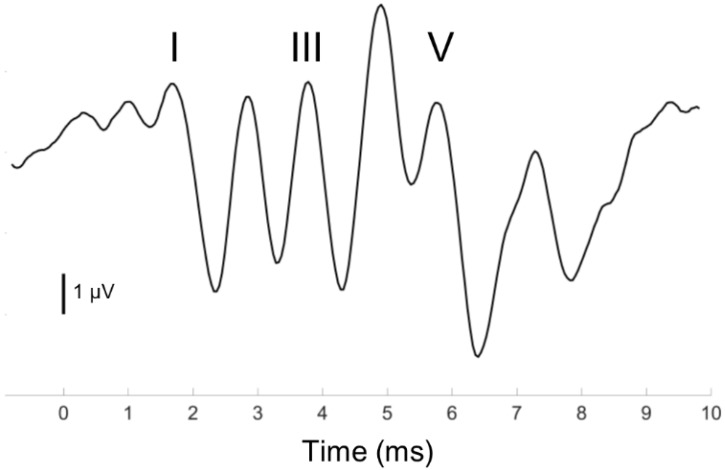
Representative Auditory Brainstem Response (ABR) evoked by a brief, click stimulus presented at 6.9/second with Waves I, III, and V labeled. Each wave is the result of synchronous neural firing from distinct neural populations in the subcortical auditory system.

Previous research on sensorineural auditory processing and reading development has primarily employed complex acoustic stimuli, such as consonant-vowel clusters, to derive electrophysiological measures of auditory processing [40,71]. However, speech-evoked neural responses likely reflect system-wide activation from subcortical and cortical auditory generators that renders their anatomical origins difficult to interpret [72,73,74,75]. By contrast, scalp-recorded ABRs to 100-microsecond click stimuli have more clearly delineated neural generators [76]. When plotted in the time domain, the click ABR has a distinct morphology consisting of five waves which generally occur over the first ~10 ms post-stimulation (Figure 1). Waves I, III, and V, the most robust of the ABR waves, originate from activity of the auditory nerve (I), dorsal cochlear nucleus (III), lateral lemniscus (V) and the inferior colliculus (V) in response to the onset of sound [76]. Inter-peak latencies (e.g., I–III, I–V) provide measures of central conduction time that corrects for differences in peripheral function [77].

While the click ABR is widely used as a clinical measure for hearing screening, the response is surprisingly underused in research on musical training. To our knowledge, only one study has directly examined links between musical training and the click ABR. This study found that pop and rock musicians exhibited faster neural responses (i.e., shorter wave latencies of the ABR) to a click stimulus relative to non-musicians [35]. Using the current paradigm, we also aimed to contribute to this nascent area of research.

In the present study, three stimulus presentation rates were used to record click ABRs at different timescales of auditory processing. These presentation rates were selected to reflect distinct, theoretical speech units, such as syllabic (e.g., 6.9 Hz) and phonemic (e.g., 31.25, 61.5 Hz) rates of temporal processing [20,70,78]. Compared to speech-evoked neural responses, recording the click ABR at varying stimulus presentation rates affords more control in (a) determining *which* generators in the subcortical auditory system may relate to reading subskills and musical history and (b) teasing apart the timescales of auditory processing thought to underlie speech perception and reading development (i.e., phonemic vs. syllabic rates).

Adopting the procedure of Skoe, Brody, and Theodore (2017) [31], the ABR protocol consisted of 100-microsecond rarefaction clicks presented at 80 dB SPL in the right ear of each participant using the Biological Navigator Pro system (Natus, Inc., Pleasanton, CA, USA). Three stimulus presentation rates were used—6.9, 31.25, and 61.5 Hz—to tap either syllabic or phonemic timescales of temporal processing [20,70,78]. Following conventional procedures, a three-electrode vertical, ipsilateral montage was used to record the far-field ABR from the scalp of participants: the active (non-inverting) electrode was located at Cz, the reference (inverting) electrode was placed on the right ear lobe (A2), and the ground electrode was placed on the forehead. Neural responses were digitized at a 24-kHz sampling rate, and filtered online from 100 to 1500 Hz. Artifacts were detected and removed online, using a signal threshold of ±23.8 microvolts. The final waveform used to derive all ABR indices was averaged online using 2000 artifact-free trials. ABR waves I, III, and V were visually identified by the experimenter before additional ABR indices (e.g., inter-peak latencies) were derived. In instances when the morphology of the ABR was unclear, two experienced ABR researchers confirmed the location of the ABR waves.

Recent work suggests that the noise exposure induced by musical training can affect cochlear function, even in individuals with clinically-normal hearing [79,80]. To account for individual differences in cochlear function, the I–III and I–V inter-peak latencies (IPLs) were calculated from the averaged ABR waveform by subtracting the absolute wave latencies of wave I from wave III, and wave I from wave V, respectively. Here, relatively longer IPLs indicate slower neural conduction time, likely resulting from decreased myelination [81], while relatively shorter IPLs reflect faster neural conduction time in central auditory structures. Additionally, as previous work has linked the repeatability of auditory-evoked potentials to reading skills [17,27], a measure of ABR response consistency (RC) for each of the three presentation rates was calculated. The RC was derived by binning the full ABR recording into smaller, 1000-trial epochs over a period of 1–8 ms and computing a correlation coefficient across the bins: a greater coefficient suggests that participants’ ABRs were more repeatable across the entire recording session [17,27,82].

### 2.2. Standardized Reading Battery

Participants completed a behavioral battery that assessed non-verbal intelligence (IQ), phonological awareness, and rapid naming (Table 1). First, we administered the Test of Nonverbal Intelligence TONI-3 to control for participants’ non-verbal IQ in our analyses. Then, we administered four reading standardized reading tests to assess specific phonological-awareness and rapid-naming subskills. In addition to administering tests for the purpose of assessing reading subskills, we also selected tests that theoretically reflect overlapping task demands with music [47,50,83]: for example, the Comprehensive Test of Phonological Processing (CTOPP) Blending Words requires participants to combine discrete speech units together to form a full, spoken word, a skill analogous to learning to combine smaller musical units (e.g., musical notes) to form larger structures (e.g., a musical melody). Tapping a similar skill, the CTOPP Elision Task requires participants to actively manipulate speech sounds by deconstructing a larger linguistic unit (e.g., a word) into its constituent parts (e.g., a syllable). We also included the CTOPP Non-Word Repetition Task to as a third measure of phonological awareness, which we argue emulates the call-and-response style of training that musicians and vocalists often undergo. During the task, participants are presented with audio recordings of non-words and then asked to immediately repeat them aloud. Finally, the Rapid Automatized Naming (RAN) subtests require participants to quickly decode single letter, digits, or both—a process arguably similar to decoding other symbolic representations of sound, such as music notation [52].

### 2.3. Music Questionnaire

A music-training questionnaire, modeled from Slater & Kraus (2016), was administered to all participants [84]. For our analyses, four variables captured participants’ musical training histories: the minimum age that participants started musical training, the total years of music training on their primary instrument, the self-reported maximum proficiency across all reported instruments, and the number of years since engaging in music training. These measures enabled us to study the variability in adult readers’ music-training histories and relate this variability to reading subskills in a continuous manner.

## 3. Results

### 3.1. Statistical and Analysis Software

Analyses were conducted using the SPSS Statistics version 25 (IPM, Armonk, NY, USA) software and the R statistical programming environment. All repeated-measures analyses were conducted using the SPSS Statistics version 25 environment, while descriptive statistics, principal components analysis, correlational analyses, and *p*-value corrections were conducted in the R programming language (version 3.3.1) using R Studio version 1.1.423 (R Core Team, Boston, MA, USA).

### 3.2. Participants

Adult participants (*n* = 25; female = 16) aged 19–22 years (mean age = 20.12 years, SD = 1.20 years) who completed tests of non-verbal IQ, phonological awareness, and rapid naming; an ABR protocol; and the music questionnaire were considered for analysis. Participants were recruited from a university campus in response to an ad posted to a campus message board for a study on auditory processing. Descriptive statistics, presented in Table 2, Table 3 and Table 4, were calculated for measures of reading subskills, music-training history, and sensorineural auditory processing (e.g., ABR indices). All participants reported no personal history of reading disorders, except one participant who reported a current diagnosis of attention-deficit hyperactivity disorder (ADHD) and a childhood history of dyslexia. All methods and protocols were approved by the Institutional Review Board (IRB) at the University of Connecticut. Prior to their participation, written informed consent was ascertained from all of the volunteers. All participants who were run through the ABR protocol exhibited otoscopy in the clinically normal range and normal bilateral air conduction thresholds ≤20 dB HL for octaves from 250 to 8000 Hz.

### 3.3. Reading Subskills and Non-Verbal IQ

Overall, participants exhibited a large degree of variability in phonological and rapid-naming skills, but fell within the normal range, mean ± 2 SDs (Table 2). (However, we note that the RAN tests are normalized for participants up to 18 years of age.) Descriptive statistics are presented in Table 2 for the standard scores on each non-verbal IQ and reading test. All participants fell within the normal range for non-verbal IQ, except one participant who had a standard score = 77. This participant, however, performed within the normal range on all reading subtests, and, thus, was included in the analysis. To assess whether the sample differed from the clinical mean of non-verbal IQ scores, a one-sample Welch’s *t*-test was performed on the mean standardized TONI scores (non-verbal IQ). Our mean score (102.3) did not differ significantly from the standardized mean score of 100, t(23) = 0.98, *p* = 0.33, 95% CI [97.49, 107.27]. One subject did not complete the TONI non-verbal IQ test due to experimenter error.

### 3.4. Auditory Brainstem Response

To confirm that our sample met the criteria of having clinically normal ABRs, the absolute wave-V latencies for the 31.25 Hz condition were compared against a large normative database for wave V latency for this presentation rate [85]. Our mean wave-V latency (31.25 Hz presentation rate) of 5.75 ms was not significantly different from the published norm of 5.69 ms, t(24) = 1.39, *p* = 0.18, 95% CI [5.66, 5.83], using a one-sample Welch’s *t*-test. Descriptive statistics for the absolute latencies, RC, and IPL measures are presented in Table 3 and Table 4.

### 3.5. Music Training Histories

Participants’ musical histories were found to be diverse, with 23 of the 25 participants reporting a history of formal musical training, leaving only 2 participants with no formal music background. Moreover, 16 participants reported a history of musical training for more than one instrument. Descriptive statistics for the musical training variables are presented in Table 5, which include the minimum age at which participants began music training, the total years of musical training on participants’ primary instrument, the number of years since music training, and the self-reported musical proficiency across all instruments on a 1-to-10 Likert scale. Over two-thirds of participants reported instrumental training as their primary mode of musical training (*n* = 19), while the remaining subjects reported vocal training (*n* = 4) as their primary mode of musical training. To assess the covariance among the music variables, Pearson’s R was calculated between all measures. Participants’ minimum age at which participants began correlated significantly with the remaining music-training variables: total years of music training, r(21) = −0.76, *p* < 0.001; the proficiency across all instruments, r(21) = −0.65, *p* < 0.001; and the years since musical training, r(21) = 0.46, *p* = 0.027, suggesting that participants who began playing music earlier, trained for longer, more recently, and reached a higher level of musical proficiency. Additionally, total years of training was related to the years since musical training, r(21) = −0.67, *p* < 0.001 and musical proficiency, r(21) = 0.76, *p* < 0.0001, suggesting that participants with more musical training also had more recent training and reached higher levels of musical proficiency. Finally, the years since musical training was related to participant’s proficiency across all instruments, r(21) = −0.80, *p* < 0.0001, indicating that more recent musical training was associated with higher levels of musical proficiency. 

### 3.6. ABR: Repeated-Measures Analyses

Previous work suggests that the absolute latencies of ABR waves gradually increase as a function of stimulus presentation rate [31,86,87]. Before deriving I–III and I–V IPLs, we aimed to ascertain the expected effect of stimulus presentation rate on the ABR latencies. Here, we implemented two Repeated-Measures Analysis of Variance (RMANOVAs) for wave III and wave V latencies, respectively. In the first RMANOVA, wave-V latencies were included as the criterion variable with stimulus presentation rate as a factor. Mauchly’s Test of Sphericity indicated that the assumption of sphericity had not been violated, χ^2^(2) = 0.929, *p* = 0.63. The RMANOVA revealed a significant main effect of stimulus rate on wave-V latencies, F(2, 48) = 65.94, *p* < 0.001, partial eta-squared = 0.73. In the second RMANOVA, wave-III latencies were included as the criterion variable with stimulus presentation rate as a factor. Mauchly’s Test of Sphericity indicated that the assumption of sphericity had been violated, χ^2^(2) = 11.67, *p* = 0.003, and therefore, a Greenhouse-Geisser correction was used. There was a significant effect of stimulus rate on wave-III latencies, F(1.430, 34.320) = 52.934, *p* < 0.001, partial eta-squared = 0.69.

Next, we assessed whether the effect of rate carried over to our derived IPL measures. Here, we implemented two RMANOVAs for I–III IPLs and I–V IPLs, respectively. In the first RMANOVA, I–V IPLs were included as the criterion variable with stimulus presentation rate as a factor. Mauchly’s Test of Sphericity indicated that the assumption of sphericity had not been violated, χ^2^(2) = 0.969, *p* = 0.70. The RMANOVA revealed a significant main effect of stimulus rate on I–V IPLs, F(2, 48) = 16.62, *p* < 0.001, partial eta-squared = 0.41. In the second RMANOVA, I–III IPLs were included as the criterion variable with stimulus presentation rate as a factor. Mauchly’s Test of Sphericity indicated that the assumption of sphericity had been violated, χ^2^(2) = 6.84, *p* = 0.033, and therefore, a Greenhouse-Geisser correction was used. There was a significant effect of stimulus rate on I–III IPLs, F(1.591, 38.182) = 4.72, *p* = 0.021, partial eta-squared = 0.164.

Collectively, these analyses suggest that stimulus presentation rate influenced both the absolute latencies of the ABR and the subsequent IPLs derived from the final waveform, albeit to slightly different degrees, given the relatively stronger effect sizes for the absolute latencies than the IPLs.

### 3.7. Principal Components Analysis: Dimensionality Reduction for Sensorineural Auditory Processing, Reading-Related Skills, and Musical Training Measures

Inflating the type-I error rate is a statistical concern when analyzing high-dimensional datasets. To reduce the number of statistical comparisons, we implemented principal component analysis (PCAs) separately for the standardized scores on the reading subtests (Table 6), ABR indices (Table 7), and musical training (Table 8) variables. PCA is a dimensionality-reduction technique that produces orthogonal principal components. The relationship between each variable and the principal component is determined by its factor loading, a standardized coefficient scaled between −1 to 1. Negative values suggest that variables track negatively with the component, while positive values suggest that variables track positively with the component. Here, we used PCA to derive a reduced set of variables (e.g., principal components) related to reading subskills, sensorineural auditory processing, and musical training that were then subjected to further correlational and partial correlation analyses. Prior to the PCA, all variables were mean-centered and scaled to standardize the variables. The PCA was implemented with rotated variables using the prcomp function from the stats R package.

#### 3.7.1. PCA: Reading Tests

The PCA for rapid-naming and phonological-awareness skills produced two factors that explained 68% of the variance in standardized reading scores, with the first factor explaining 46% of the variance and the second explaining 23% of the variance. Both the RAN subtests and CTOPP subtests loaded negatively on factor 1, suggesting that participants with higher reading factor-1 scores have lower standardized RAN and CTOPP scores. Moreover, CTOPP subtests largely loaded on to factor 2 with negative loadings, while the RAN subtests loaded positively onto factor 2. This suggests that participants with higher reading factor-2 scores have higher standardized RAN scores, but lower CTOPP standardized scores.

#### 3.7.2. PCA: ABR Indices

The PCA for sensorineural auditory processing yielded two factors that explained 65% of the variance in ABR indices, with the first factor explaining 45% of the variance and the second factor explaining 20% of the variance. ABR indices, in general, loaded positively onto the first factor, with measures of neural conduction times (e.g., IPLs) loading more strongly than measures of neural consistency (e.g., RC). Thus, participants with higher ABR factor-1 scores had slower neural conduction times (i.e., larger IPLs), while participants with lower ABR factor-1 scores had faster neural conduction times (i.e., smaller IPLs). Interestingly, the PCA produced loadings that were similar in magnitude across the three click presentation rates, indicating, in part, that although IPLs prolong as the rate increases, that the ABRs to these three rates pattern together and may therefore reflect a common physiological mechanism. Additionally, both I–III and I–V IPLs loaded on to ABR factor 1 to a similar degree, indicating that I–III and I–V IPLs pattern together, and, therefore, this factor should be interpreted a global measure of auditory processing within the brainstem. Finally, ABR indices, in general, loaded negatively on ABR factor 2, with neural consistency measures loading more strongly. This suggests participants with higher ABR factor-2 scores had less consistent ABRs.

#### 3.7.3. PCA: Musical Training Variables

The PCA for the musical-training variables yielded a single factor that captured 76% of the variance in musical experience, suggesting that this factor served as a global musical-training variable. Minimum age of musical training and years since musical had positive loadings on factor 1, while max proficiency and total years of musical training had negative loadings on factor 1. Thus, participants with higher musical-training factor-1 scores had less global musical experience, with less musical profanely, less total years of musical training, less recent musical training, and a later age of musical training onset. 

### 3.8. Correlation Analyses between Principal Components

Next, in three separate analyses to address our three primary questions, we investigated relations between sensorineural auditory processing, reading subskills, and a history of music training by computing Pearson correlations between components derived from PCA. We also controlled for multiple comparisons by implementing the Benjamini & Hochberg (BH) procedure to control the false-discovery rate (FDR) across all comparisons with an alpha level set at 0.05 [88]. The BH procedure was implemented as a global correction across all three analyses to produce corrected probability values. Finally, partial correlations were computed with participants’ age in days and the standardized TONI scores entered as a covariate to control participants’ age and non-verbal IQ. In cases involving missing data, pair-wise deletion was used.

#### 3.8.1. Question 1: Is sensorineural Auditory Processing Related to Phonological and Rapid Naming Skills in Adulthood?

**Figure 2:**
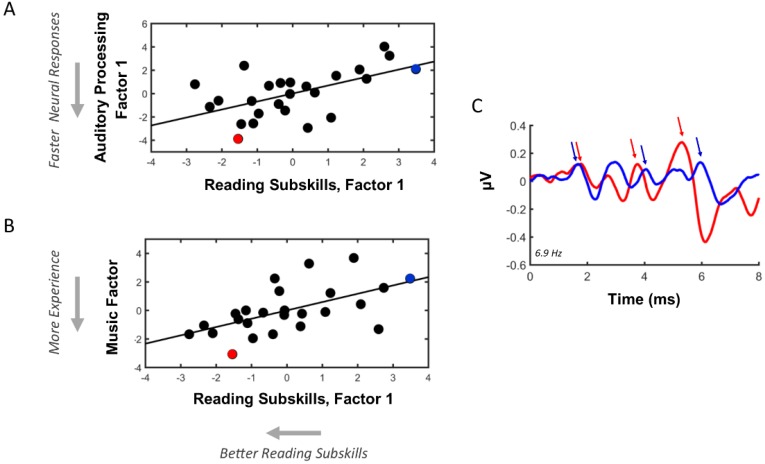
Summary of relations between sensorineural auditory processing, reading subskills, and musical experience. (**A**) Scatter plot between reading and auditory brainstem response (ABR) factors derived from principal component analysis (PCA). Faster neural conduction times (i.e., lower ABR factor-1 scores) are related to stronger rapid-naming skills (i.e., lower reading factor-1 scores). To aid in the visual interpretation of the relationship, arrows located on the axis denote the direction of factor loadings on each principal component. (**B**) Scatter plot between reading and musical training factors derived from PCA. More musical training (i.e., lower musical training factor scores) is associated with stronger rapid-naming skills (i.e., lower reading factor-1 scores). (**C**) Representative ABRs from a participant (red) with stronger reading subskills, faster neural conduction times, and more musical experience relative to a participant (blue) with weaker reading subskills, slower neural conduction times, and less musical experience. The participant in red had 20 years of total musical training beginning at age 2 years. The participant in blue had 3 total years of musical training beginning at age 15. Note that the latencies of the first ABR peak (e.g., ABR wave I) align across the two participants, while the later waves begin to diverge, demonstrating that the participant marked in blue exhibited slower neural conduction times. In panels A and B, the scatter dots marked in red and blue reflect these two participants.

First, we investigated relations between sensorineural auditory processing and reading subskills by computing Pearson correlations between the ABR and reading factors derived from PCA (Figure 2A, Table 9). ABR factor 1 was negatively related to the reading factor 1, r(23) = 0.56, *p* = 0.003, corrected *p*-value = 0.014, suggesting that participants with faster neural conduction times had stronger rapid-naming skills. Moreover, this relationship held after controlling for participants’ age, pr(22) = 0.56, *p* = 0.002, and participants’ non-verbal IQ (i.e., TONI scores), pr(21) = 0.59, *p* = 0.0008.

#### 3.8.2. Question 2: Do Differences in Music-Training History Relate to Phonological and Rapid Naming Skills in Adulthood?

Next, we investigated relations between musical training and reading subskills. Pearson correlations were computed between musical training and reading factors derived from PCA (Figure 2B, Table 10). Musical training factor 1 was found to positively relate to the reading factor 1, r(21) = 0.58, *p* = 0.003, corrected *p*-value = 0.014, suggesting that earlier musical training, more total years of musical training, more recent musical, and an early age of the onset of musical training was associated with stronger rapid-naming skills. This relationship held even after accounting for participants’ age, pr(20) = 0.61 *p* < 0.001, and participant’s non-verbal IQ (i.e., TONI scores), pr(19) = 0.54, *p* = 0.004.

While musical training was related reading factor 1, musical training factor 1 was not significantly related to reading factor 2, r(21) = 0.21, *p* = 0.33, corrected *p*-value = 0.44, suggesting that musical training was not related to phonological-awareness skills in this study sample.

#### 3.8.3. Question 3: Do Differences in Music-Training History Relate to Sensorineural Auditory Processing?

Finally, we investigated relations between sensorineural auditory processing and a history of musical training. Pearson correlations were computed between the musical training and ABR factors derived from PCA (Table 11). Musical training factor 1 was moderately related to ABR factor 1, suggesting that more musical experience was related to faster neural conduction times, though this relationship was not significant after controlling for the FDR, r(21) = 0.42, *p* = 0.044, corrected *p*-value = 0.119. Controlling for non-verbal IQ also produced a moderate relationship, pr(19) = 0.44, *p* = 0.032, while the relationship weakened after accounting for participants’ age in days, pr(20) = 0.38, *p* =0.066.

Moreover, musical training factor 1 was not significantly related to ABR factor 2, r(21) = −0.18, *p* = 0.42, corrected *p*-value = 0.48, suggesting that musical training was not related to neural response consistency. While musical experience was moderately related to sensorineural processing, this relationship was not significant after accounting for the FDR and participants’ age.

## 4. Discussion

### 4.1. Summary of Findings

The current study investigated relationships between sensorineural auditory processing, musical training, and reading subskills in a sample of young adult readers. In an attempt to expand upon previous work regarding relations between musical training, auditory processing, and reading subskills in childhood, here, we employed a series of principal component analyses to derive general factors of adult readers’ sensorineural auditory processing, reading subskills, and musical experience. These factors were then correlated to elucidate relations between sensorineural auditory processing, reading subskills, and musical experience in adulthood. Consistent with previous work that suggests faster neural responses to sound are associated with better reading skills in children [24], we found that faster neural conduction times in central auditory structures were associated with stronger reading subskills. In particular, we found that neural conduction times were largely related to rapid-naming skills, with faster neural conduction times related to better performance. These relationships did not appear to be timescale-specific (i.e., the ABR factor 1 contained similar loadings for IPLs derived from ABRs recorded at syllabic and phonemic rates), likely reflecting the importance of multiple timescales of auditory processing for reading development [5,11,20]. Next, we found that participants with more musical training experience had stronger rapid-naming skills. This finding is consistent with previous work reporting that musical competence and musical training are related to reading subskills [25,47,48,49,50,51,53,55,67]. While we found a relationship between musical experience and rapid naming [49,54], unlike previous work, we did not find that musical experience was related to phonological awareness [50,51,67].

Further, we investigated whether sensorineural auditory processing related to musical training history: while a moderate relationship was found, suggesting that earlier, more recent, longer, and more proficient musical training was related to faster neural conductions times, this relationship was not significant after controlling the false discovery rate (FDR). This is likely due to the limitations of a small sample and low statistical power to detect small-to-moderate effect sizes. However, the moderate trend is nevertheless consistent with work that suggests adult musicians have faster neural responses and more myelinated neural pathways [35,43,89,90,91,92].

While several relationships emerged between neural conduction times and reading subskills, unlike previous work, we did not find evidence that the repeatability of auditory-evoked neural responses (i.e., neural response consistency) was related to reading subskills [17,29]. This may reflect differences in auditory-stimulus complexity [82] or differences in reading competency between our study sample and previous investigations. Indeed, past work probed neural response consistency in relation to developmental dyslexia and autism spectrum disorder, whereas we examined relationships between reading ability and the repeatability of sensorineural processing in a largely unimpaired, adult population.

### 4.2. Limitations and Future Work

While a strength of the current study was the ability to investigate relationships between sensorineural auditory processing, music training, and reading skills within a single adult sample, a major limitation of the current study was the small number of participants (*n* = 25). Furthermore, the limited sample prevented us from directly testing whether sensorineural auditory processing mediates the relationship between musical training and reading subskills skills [4]. Future work, using larger samples of adult readers, should investigate whether sensorineural auditory processing mediates musical training history and reading-related skills with path analysis or structural-equation modeling (SEM) [25]. Moreover, many of the participants in our sample reported a history of musical experience, precluding us from drawing general conclusions about musicians and non-musicians. Future would could include more untrained participants to conduct group-level analyses, while also taking a continuous approach for specific music-training variables [93,94,95]. Additionally, inclusion of non-musically trained mature readers would enable us to assess whether there is a threshold effect of musical training (i.e., a minimum amount needed to engender reading benefits) or a ceiling effect (i.e., an overall limit on reading benefits that music training may confer), as has been implicated in developmental work (Gordon et al., 2015). Another limitation of our sample is that we are likely tapping a limited range of reading subskills than is truly reflected at the population level. Here, we recruited entirely from a college-aged population at a major university. However, even within our sample, we observed variability across reading subskills, reflecting average-to-above-average reading-related skills. Relatedly, while we investigated specific reading subskills in this study, specifically reading subskills that had previously been linked to musical training and musical aptitude in children, we did not include a measure of global reading ability (e.g., reading fluency). Future work could examine whether the relationships observed here among reading subskills translate to more global measures of reading. Moreover, we did not account for participants’ history of reading experience [96], i.e., we did not explore the possibility that variability in reading subskills could arise from variability in reading experience: participants who read less are less likely to become skilled readers [96]. Thus, those who are musically proficient may also, on average, read more than those who are less musically proficient. Nevertheless, there is some evidence that music skills are still related to reading skills, even when controlling for reading experience. For instance, Corrigall & Trainor (2011) found that the length of music training in a cohort of 6- to 9-year-old children was related to reading comprehension, even when the numbers of hours spent reading was controlled for.

Additionally, the present study probed only one putative pathway of music-reading transfer: sensorineural auditory processing. Consequently, we did not explicitly account for higher cognitive abilities that might relate to both reading ability and music training (e.g., working auditory memory, attention) [97,98,99,100]. While our study did not use tests to probe these higher level abilities beyond non-verbal IQ, previous work has shown that they are related to a history of musical training and music perception [101,102,103]. And, finally, we did not directly assess musical skills (e.g., rhythm and pitch abilities)—future work could assess rhythm and pitch skills in adult readers [104], and associate those with a history of music training, auditory processing, and reading-related skills [15]. Moreover, we did not account for the socio-economic status (SES) of our participants, a potential confounding variable, as previous work suggests that SES is related to auditory brainstem function, musical training, and reading skills [105,106].

In our experimental paradigm, we employed a broadband click stimulus presented at different rates to isolate the different timescales of temporal processing important for speech perception and reading development. However, our use of a broadband click stimulus to evoke neural responses to sound may not adequately reflect the hierarchically nested timescales of natural speech. Expanding our stimulus set to include speech stimuli with formant transitions or pitch contours might be a more ecologically valid way of assessing the neural encoding of speech dynamics across different timescales, e.g., [40,71]. This would allow us to make a direct comparison regarding the sensitivity of the click ABR and speech-evoked neural responses to predict reading behaviors in musically trained populations.

### 4.3. Investigating Musical Training, Senorineural Processing, and Reading Across the Lifespan

While work on sensorineural auditory processing, musical training, and reading development has typically been studied emerging readers, it is unknown whether musical training ultimately alters reading trajectories and reading outcomes. Indeed, previous work has often employed cross-sectional or longitudinal designs to investigate music-reading relations during childhood, though never extending to adulthood. The current study suggests that relations between sensorineural auditory processing, musical training, and reading subskills may be observable in adult readers.

**Figure 3:**
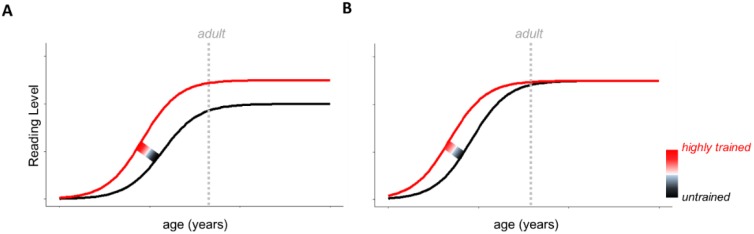
Two Theoretical Reading-Acquisition Functions (**A**) Overall Enhancement: Children who are receiving musical training display an increased, overall enhancement in reading subskills, and, consequently, global reading ability that persists into adulthood. (**B**) Developmentally Early Enhancement: Children who are receiving musical training display a faster rate in the acquisition of reading subskills, and, consequently, global reading ability than children who do not. However, non-musically trained children catch up to their musically trained peers in adulthood. For both (**A**,**B**), the effect of musical training is predicted to be graded, with more extensive musical training associated with better auditory processing and in turn higher reading subskills and global reading levels. In both models, the gradient effect is predicted to be non-linear: a minimum amount of training might be required to see an effect of music training on reading, and there might be a ceiling effect, whereby additional training above a certain threshold no longer engenders additional reading-related benefits.

To help guide future research programs, we conclude by proposing two, theoretical reading-acquisition models, informed by the extant developmental literature, to generate developmental predictions on how musical training might interact with sensorineural auditory processing and reading development (Figure 3A,B). Importantly, both models reflect the current findings that suggest a reading-subskill advantage for children with higher degrees of musical experience and musical aptitude [5,25,47,48,49,50,51,53]. However, the models differ in their predictions regarding the influence of musical training on reading beyond the period of childhood. The first model posits that, on average, musically trained individuals have more robust reading subskills, and, perhaps, better global reading ability, by virtue of having enhanced sensorineural auditory processing. In this model, reading-related advantages are predicted to persist into adulthood (Figure 3A). The second model posits that musical training increases the initial rate of literacy (i.e., the acquisition of reading subskills, and, later, global reading ability), but that children with less training or no training eventually converge to their musically trained peers at a later stage of reading development (Figure 3B). In the second model, the advantages associated with musical training are thought to bootstrap only the earliest phases of reading development (i.e., childhood).

**Figure 4:**
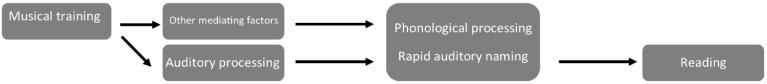
One Putative Pathway by which Musical Training May Strengthen Reading-Related Skills. We propose that musical training bolsters reading subskills through sensorineural auditory processing [4,18]. However, there are other likely pathways through which a music advantage may occur (e.g., auditory working memory, attention) [4].

Similar to previous models on music and reading, both of these models operate from the assumption that one pathway by which musical training enhances reading subskills, and, consequently, global reading ability, is by bolstering sensorineural auditory processing (Figure 4) [4,18]. In a statistical framework, this would suggest that sensorineural auditory processing is a mediating variable between musical training and reading subskills [25]. Additionally, music training is predicted to interact with reading development in a graded fashion, with more extensive training associated with greater reading outcomes across development. Lastly, we note that while musical training is suggested here to provide a reading advantage, it is likely that other experiential factors also enhance reading skills. As such, the models do not claim that musical training is the only method for bolstering reading subskills, nor does it suggest that untrained readers will develop reading or language deficits.

While previous work has revealed important relationships between musical experience, auditory processing, and reading subskills in samples of emerging readers, cross-sectional data from emerging readers alone cannot tease apart these two competing models, nor can data obtained from longitudinal studies that fall short of adulthood. While the current study may suggest that musical experience, sensorineural auditory processing, and reading subskills are related in adulthood, we argue that the ideal methodology to examine these hypotheses is a longitudinal study tracking reading outcomes, beginning in childhood and continuing through adulthood, with children who are undergoing musical training and a group of children who are undergoing a comparable, control activity (e.g., art lessons). Future work could, thus, employ longitudinal or fine-grained cross-sectional designs to investigate how musical experience interacts with sensorineural processing and reading development across the lifespan.

## 5. Conclusions

In summary, the current study found that reading subskills (e.g., rapid naming) related to both sensorineural processing and musical experience in a sample of adult readers. Here, we found that adult participants with faster neural conduction times and more musical experience had stronger rapid-naming skills. These findings are similar to relationships between musical experience, sensorineural auditory processing, and reading subskills that have been reported in children. We concluded by proposing two lifespan models of music-reading transfer to help guide future research programs on musical training, sensorineural auditory processing, and reading subskills.

## Figures and Tables

**Table 1:** Behavioral Reading Battery. Descriptions of the individual tests used to probe participants’ non-verbal intelligence (IQ) and reading subskills in the present study. Specific reading subskills included phonological awareness and rapid naming.

Test	Subtest	Description
Test of Non-Verbal Intelligence (TONI-3)	Non-Verbal IQ	Untimed task. Select an illustration from a set of illustrations to complete a visual puzzle
Comprehensive Test of Phonological Processing (CTOPP)	Elision	Untimed task. Say part of a word after saying the whole word (e.g., Say the word ‘spider’. Now say ‘spider’ without saying ‘der’)
	Blending Words	Untimed task. Combine sounds of a word to form one word (e.g., ‘can’ + ‘dy’ = ‘candy’)
	Non-Word Repetition	Untimed task. Repeat back a list of non-words
RAN (Rapid Automatized Naming)	RAN Numbers	Timed task. Read aloud a list of numbers as quickly as possible
	RAN Letters	Timed task. Read aloud a list of letters as quickly as possible
	RAS 2-set	Timed task. Read aloud a list that contains both numbers and letters as quickly as possible

**Table 2:** Descriptive statistics for the reading battery and non-verbal IQ test. Means, standard deviations (SD) in parenthesis, and ranges of the standard scores for each test are reported.

Test	Mean (SD)	Range of Standard Scores
TONI	102.3 (11.69)	77–130
CTOPP Elision	10.08 (1.35)	7–12
CTOPP Blending Words	11.16 (1.72)	8–13
CTOPP Nonword Repetition	9.8 (1.94)	7–14
RAN Numbers	110.1 (4.53)	102–117
RAN Letters	108.6 (4.97)	98–117
RAN 2 Set	112.1 (6.11)	100–125

**Table 3:** Descriptive statistics of auditory brainstem response (ABR) absolute latencies and Response Consistency (RC). Means and standard deviations (parenthesis) or ABR absolute latencies and the RC measures. Here, the response consistency (RC) reflects the repeatability of the click ABR over the duration of the recording session (i.e., a higher RC means a more consistent ABR).

Rate	Wave III	Wave V	Response Consistency (RC)
6.9 Hz	3.85 (0.18) ms	5.69 (0.25) ms	0.80 (0.18) Pearson’s r
31.25 Hz	3.94 (0.17) ms	5.75 (0.21)ms	0.73 (0.20) Pearson’s r
61.5 Hz	4.04 (0.18) ms	5.96 (0.24) ms	0.71 (0.27) Pearson’s r

**Table 4:** Descriptive statistics of auditory brainstem response (ABR) Inter-Peak Latencies (IPLs). Means and standard deviations (parenthesis) or ABR inter-peak latencies for waves I–III and I–V.

Rate	I–III IPL	I–V IPL
6.9 Hz	2.21 (0.18) ms	4.04 (0.23) ms
31.25 Hz	2.26 (0.17) ms	4.07 (0.20) ms
61.5 Hz	2.30 (0.15) ms	4.22 (0.20) ms

**Table 5:** Descriptive statistics of music training (MT) variables. Means and standard deviations (SD) in parenthesis for the minimum age of musical training, total years of musical training on primary instrument, self-rated maximum proficiency, and the number of years since musical training.

Musical Training (MT) Variable	Mean (SD)	Range
Minimum Age of MT	8.17 (3.30) Years	2–15 Years
Total Years of MT	8.36 (4.91) Years	0–20 Years
Max Proficiency of MT	7.78 (2.02)	3–10
Years Since MT	1.17 (1.75) Years	0–5 Years

**Table 6:** Principal component analysis (PCA) of reading subtests. The factors and factor loadings of the PCA computed over the standard scores of the Comprehensive Test of Phonological Processing (CTOPP) and Rapid Automatized Naming (RAN) subtests. The PCA yielded two factors that explained 68% of the total variance in standardized test scores. Factor 1 had negative loadings for the both the CTOPP and RAN subtests, albeit much stronger loadings for the RAN scores, suggesting this factor reflects primarily rapid-naming skills. Factor 2 produced moderately sized negative loadings for the CTOPP tests and smaller positive loadings from the RAN tests, suggesting that this factor largely reflects phonological-awareness skills.

	Factor I	Factor II
CTOPP Blending Words	−0.30	−0.64
CTOPP Elision	−0.25	−0.36
CTOPP Nonword Repetition	−0.21	−0.52
RAN Letters	−0.53	0.28
RAN Numbers	−0.51	0.26
RAN 2 Set	−0.51	0.21
Proportion of Variance	0.46	0.23
Culmulative Variance	0.46	0.68

**Table 7:** PCA of ABR Indices. The factors and factor loadings of the PCA computed over indices of sensorineural auditory processing derived from the averaged auditory brainstem response (ABR). The PCA yielded two factors that explained 65% of the cumulative variance in ABR indices. Factor 1 had stronger loadings for the inter-peak latency measures (IPLs), suggesting that this factor largely reflected subcortical neural conduction times. Factor 2 had stronger loadings for the response consistency (RC) measures, suggesting that this factor largely reflected the response consistency of the ABR over a single recording session.

	Factor I	Factor II
RC (6.9 Hz)	0.02	−0.65
RC (31.25 Hz)	−0.03	−0.42
RC (61.5 Hz)	−0.22	−0.49
I–V IPL (6.9 Hz)	0.44	0.05
I–V IPL (31.25 Hz)	0.39	0.14
I–V IPL (61.5 Hz)	0.41	0.10
I–III IPL (6.9 Hz)	0.38	−0.23
I–III IPL (31.25 Hz)	0.42	−0.20
I–III IPL (61.5 Hz)	0.34	−0.16
Proportion of Variance	0.45	0.20
Cumulative Variance	0.45	0.65

**Table 8:** PCA of the Musical Training Variables. The factor and factor loadings of the PCA computed over four musical training variables. The PCA yielded one factor that explained 76% of the total variance. Factor 1 had moderate-to-large loadings for all four musical training variables, suggesting that this factor served as a global musical training variable.

Factor 1	
Max Profiency	−0.53
Total Years of Musical Training	−0.52
Minum Age of Musical Training	0.47
Years Since Musical Training	0.48
Proportion of Variance	0.76

**Table 9:** Relations between ABR and Reading Factors. Pearson’s R computed among auditory brainstem (ABR) and reading subskills (e.g., RAN and CTOPP tests) using factors derived from PCA. Here, ABR factor 1 predominately reflected neural condition times (e.g., inter-peak latencies of the ABR waves I to III and wave I to V), while ABR factor 2 largely reflected neural response consistency. Reading factor 1 largely reflected rapid naming skills, while reading factor 2 primarily reflected phonological awareness skills. The correlations are presented with corrected probability values using the Benjamini & Hochberg (BH) correction to control the false discovery rate (FDR).

Reading Subtests (Factor 1)			
	Pearson’s R	*p*-Value	Corrected *p*-Value
**ABR (Factor 1)**	0.56	0.003	0.014
**ABR (Factor 2)**	0.25	0.231	0.40
**Reading Subtests (Factor 2)**			
	**Pearson’s R**	***p*-Value**	**Corrected *p*-Value**
**ABR (Factor 1)**	−0.01	0.96	0.96
**ABR (Factor 2)**	−0.24	0.25	0.40

ABR factor 2 was not significantly related to reading factor 1, r(23) = 0.25, *p* = 0.23, corrected *p*-value = 0.40, suggesting that neural response consistency was not related to rapid-naming skills. Additionally, neither ABR factors 1 or 2 significantly related to the reading factor 2: ABR factor 1 and reading factor 2, r(23) = −0.01, *p* = 0.96, corrected *p*-value = 0.96; and ABR factor 2 and reading factor 2, r(23) = −0.24, *p* = 0.25, corrected *p*-value = 0.40. This suggests that neither neural conduction times nor neural response consistency related to phonological-awareness skills in this study sample.

**Table 10:** Relations between Musical Training (MT) History and Reading Tests. Pearson’s R computed musical training and reading factors derived from PCA. Here, musical training factor 1 reflected a global measure of musical experience. Reading factor 1 largely reflected rapid-naming skills, while reading factor 2 primarily reflected phonological-awareness skills. The correlations are presented with corrected probability values using the Benjamini & Hochberg (BH) correction to control the false discovery rate (FDR).

Reading Subtests (Factor 1)			
	Pearson’s R	*p*-Value	Corrected *p*-Value
**Musical Training (Factor 1)**	0.58	0.003	0.014
**Reading Subtests (Factor 2)**			
	**Pearson’s R**	***p*-Value**	**Corrected *p*-Value**
**Musical Training (Factor 1)**	0.21	0.33	0.44

**Table 11:** Relations between Musical Training History and Sensorineural Auditory Processing. Pearson’s R computed between musical training and auditory brainstem response (ABR) factors. Here, musical training factor 1 reflected a global measure of musical experience. ABR factor 1 predominately reflected neural condition times (e.g., inter-peak latencies of the ABR waves I to III and wave I to V), while ABR factor 2 largely reflected neural response consistency. The correlations are presented with corrected probability values using the Benjamini & Hochberg (BH) correction to control the false discovery rate (FDR).

ABR (Factor 1)			
	Pearson’s R	*p*-Value	Corrected *p*-Value
**Musical Training (Factor 1)**	0.42	0.044	0.119
**ABR (Factor 2)**			
	**Pearson’s R**	***p*-Value**	**Corrected *p*-Value**
**Musical Training (Factor 1)**	−0.18	0.42	0.48

## References

[1] Bradley L., Bryant P.E. (1983). Categorizing sounds and learning to read—A causal connection. Nature.

[2] Bentin S. (1992). Phonological Awareness, Reading, and Reading Acquisition: A Survey and Appraisal of Current Knowledge. Haskins Lab. Status Rep. Speech Res..

[3] Manis F.R., Seidenberg M.S., Doi L.M. (1999). See Dick RAN: Rapid Naming and the Longitudinal Prediction of Reading Subskills in First and Second Graders. Sci. Stud. Read..

[4] Tallal P., Gaab N. (2006). Dynamic auditory processing, musical experience and language development. Trends Neurosci..

[5] Huss M., Verney J.P., Fosker T., Mead N., Goswami U. (2011). Music, rhythm, rise time perception and developmental dyslexia: Perception of musical meter predicts reading and phonology. Cortex.

[6] Ramus F. (2002). Language discrimination by newborns: Teasing apart phonotactic, rhythmic, and intonational cues. Annu. Rev. Lang. Acquis..

[7] Ramus F., Nespor M., Mehler J. (1999). Correlates of linguistic rhythm in the speech signal. Cognition.

[8] Tallal P., Miller S., Fitch R. (1993). Neuropsychological Bases of Speech: A case for the Preeminance of Temporal Processing. Ann. N. Y. Acad. Sci..

[9] Protopapas A. (2014). From temporal processing to developmental language disorders: Mind the gap. Philos. Trans. R. Soc. Lond. B. Biol. Sci..

[10] Holliman A.J., Wood C., Sheehy K. (2010). Does speech rhythm sensitivity predict children’s reading ability 1 year later?. J. Educ. Psychol..

[11] Tallal P. (1980). Auditory temporal perception, phonics, and reading disabilities in children. Brain Lang..

[12] McAnally K.I., Stein J.F. (1996). Auditory temporal coding in dyslexia. Proc. Biol. Sci..

[13] Boets B., Vandermosten M., Poelmans H., Luts H., Wouters J., Ghesquière P. (2011). Preschool impairments in auditory processing and speech perception uniquely predict future reading problems. Res. Dev. Disabil..

[14] Benasich A.A., Tallal P. (2002). Infant discrimination of rapid auditory cues predicts later language impairment. Behav. Brain Res..

[15] Banai K., Ahissar M. (2013). Musical Experience, Auditory Perception and Reading-Related Skills in Children. PLoS ONE.

[16] Kraus N., Anderson S. (2013). For Reading Development, Auditory Processing Is Fundamental. Hear. J..

[17] Hornickel J., Kraus N. (2013). Unstable representation of sound: A biological marker of dyslexia. J. Neurosci..

[18] Tierney A., Kraus N. (2013). Music Training for the Development of Reading Skills.

[19] Gervain J., Mehler J. (2010). Speech perception and language acquisition in the first year of life. Annu. Rev. Psychol..

[20] Goswami U. (2011). A temporal sampling framework for developmental dyslexia. Trends Cogn. Sci..

[21] Boets B., Wouters J., van Wieringen A., De Smedt B., Ghesquière P. (2008). Modelling relations between sensory processing, speech perception, orthographic and phonological ability, and literacy achievement. Brain Lang..

[22] Saffran J., Aslin R., Newport E. (1996). Statistical learning by 8-month-old infants. Science.

[23] Hornickel J., Anderson S., Skoe E., Yi H.H.-G., Kraus N. (2012). Subcortical representation of speech fine structure relates to reading ability. Neuroreport.

[24] Banai K., Hornickel J., Skoe E., Nicol T., Zecker S., Kraus N. (2009). Reading and subcortical auditory function. Cereb. Cortex.

[25] Strait D.L., Hornickel J., Kraus N. (2011). Subcortical processing of speech regularities underlies reading and music aptitude in children. Behav. Brain Funct..

[26] Hornickel J., Chandrasekaran B., Zecker S., Kraus N. (2011). Auditory brainstem measures predict reading and speech-in-noise perception in school-aged children. Behav. Brain Res..

[27] Hornickel J., Knowles E., Kraus N. (2012). Test-retest consistency of speech-evoked auditory brainstem responses in typically-developing children. Hear. Res..

[28] Hornickel J., Skoe E., Nicol T., Zecker S., Kraus N. (2009). Subcortical differentiation of stop consonants relates to reading and speech-in-noise perception. Proc. Natl. Acad. Sci. USA.

[29] Neef N.E., Müller B., Liebig J., Schaadt G., Grigutsch M., Gunter T.C., Wilcke A., Kirsten H., Skeide M.A., Kraft I. (2017). Dyslexia risk gene relates to representation of sound in the auditory brainstem. Dev. Cogn. Neurosci..

[30] Hornickel J., Zecker S.G., Bradlow A.R., Kraus N. (2012). Assistive listening devices drive neuroplasticity in children with dyslexia. Proc. Natl. Acad. Sci. USA.

[31] Skoe E., Brody L., Theodore R.M. (2017). Reading ability reflects individual differences in auditory brainstem function, even into adulthood. Brain Lang..

[32] Skoe E., Kraus N. (2013). Musical training heightens auditory brainstem function during sensitive periods in development. Front. Psychol..

[33] Tierney A., Krizman J., Skoe E., Johnston K., Kraus N. (2013). High school music classes enhance the neural processing of speech. Front. Psychol..

[34] Slater J., Tierney A., Kraus N. (2013). At-risk elementary school children with one year of classroom music instruction are better at keeping a beat. PLoS ONE.

[35] Samelli A., Carvallo R.M., de Beija C., Rabelo C., Matas C., Gomes R., Magliaro F.L. (2012). Audiological and electrophysiological assessment of professional pop/rock musicians. Noise Health.

[36] Parbery-Clark A., Anderson S., Hittner E., Kraus N. (2012). Musical experience strengthens the neural representation of sounds important for communication in middle-aged adults. Front. Aging Neurosci..

[37] Skoe E., Kraus N. (2012). A Little Goes a Long Way: How the Adult Brain Is Shaped by Musical Training in Childhood. J. Neurosci..

[38] Chandrasekaran B., Hornickel J., Skoe E. (2009). Context-dependent encoding in the human auditory brainstem relates to hearing speech in noise: Implications for developmental dyslexia. Neuron.

[39] Parbery-Clark A., Tierney A., Strait D.L., Kraus N. (2012). Musicians have fine-tuned neural distinction of speech syllables. Neuroscience.

[40] Wong P., Skoe E., Russo N. (2007). Musical experience shapes human brainstem encoding of linguistic pitch patterns. Nat. Neurosci..

[41] Bidelman G.M., Krishnan A. (2010). Effects of reverberation on brainstem representation of speech in musicians and non-musicians. Brain Res..

[42] Weiss M., Bidelman G. (2015). Listening to the Brainstem: Musicianship Enhances Intelligibility of Subcortical Representations for Speech. J. Neurosci..

[43] Musacchia G., Sams M., Skoe E., Kraus N. (2007). Musicians have enhanced subcortical auditory and audiovisual processing of speech and music. Proc. Natl. Acad. Sci. USA.

[44] Strait D.L., Parbery-Clark A., Hittner E., Kraus N. (2012). Musical training during early childhood enhances the neural encoding of speech in noise. Brain Lang..

[45] Patel A.D. (2011). Why would Musical Training Benefit the Neural Encoding of Speech? The OPERA Hypothesis. Front. Psychol..

[46] Patel A.D. (2014). Can nonlinguistic musical training change the way the brain processes speech? The expanded OPERA hypothesis. Hear. Res..

[47] Anvari S.H., Trainor L.J., Woodside J., Levy B.A. (2002). Relations among musical skills, phonological processing, and early reading ability in preschool children. J. Exp. Child Psychol..

[48] Forgeard M., Schlaug G., Norton A., Rosam C., Iyengar U., Winner E. (2008). The relation between music and phonological processing in normal-reading children and children with dyslexia. Music Percept. Interdiscip. J..

[49] David D., Wade-Woolley L., Kirby J.R., Smithrim K. (2007). Rhythm and reading development in school-age children: A longitudinal study. J. Res. Read..

[50] Peynircioglu Z.F., Durgunoglu A.Y., Uney-Kusefoglu B. (2002). Phonological awareness and musical aptitude. J. Res. Read..

[51] Degé F., Kubicek C., Schwarzer G. (2015). Associations between musical abilities and precursors of reading in preschool aged children. Front. Psychol..

[52] Ganschow L., Lloyd-Jones J., Miles T.R. (1994). Dyslexia and musical notation. Ann. Dyslexia.

[53] Goswami U., Huss M., Mead N., Fosker T., Verney J.P. (2013). Perception of patterns of musical beat distribution in phonological developmental dyslexia: Significant longitudinal relations with word reading and reading comprehension. Cortex.

[54] Woodruff Carr K., White-Schwoch T., Tierney A.T., Strait D.L., Kraus N. (2014). Beat synchronization predicts neural speech encoding and reading readiness in preschoolers. Proc. Natl. Acad. Sci. USA.

[55] Douglas S., Willatts P. (1994). The relationship between musical ability and literacy skills. J. Res. Read..

[56] Hämäläinen J.A., Salminen H.K., Leppänen P.H.T. (2013). Basic Auditory Processing Deficits in Dyslexia: Systematic Review of the Behavioral and Event-Related Potential/Field Evidence. J. Learn. Disabil..

[57] Moreno S., Friesen D., Bialystok E. (2011). Effect of music training on promoting preliteracy skills: Preliminary causal evidence. Music Percept..

[58] Habib M., Lardy C., Desiles T., Commeiras C., Chobert J., Besson M. (2016). Music and Dyslexia: A New Musical Training Method to Improve Reading and Related Disorders. Front. Psychol..

[59] Bhide A., Power A., Goswami U. (2013). A Rhythmic Musical Intervention for Poor Readers: A Comparison of Efficacy With a Letter-Based Intervention. Mind Brain Educ..

[60] Cogo-Moreira H., Brandão de Ávila C.R., Ploubidis G.B., Mari J.D.J. (2013). Effectiveness of music education for the improvement of reading skills and academic achievement in young poor readers: A pragmatic cluster-randomized, controlled clinical trial. PLoS ONE.

[61] Overy K. (2003). Dyslexia and Music: From Timing Deficits to Musical Intervention. Ann. N. Y. Acad. Sci..

[62] Rautenberg I. (2015). The effects of musical training on the decoding skills of German-speaking primary school children. J. Res. Read..

[63] Register D., Darrow A.A., Swedberg O., Standley J. (2007). The Use of Music to Enhance Reading Skills of Second Grade Students and Students with Reading Disabilities. J. Music Ther..

[64] Marie C., Magne C., Besson M. (2011). Musicians and the Metric Structure of Words. J. Cogn. Neurosci..

[65] Bishop-Liebler P., Welch G., Huss M., Thomson J.M., Goswami U. (2014). Auditory temporal processing skills in musicians with dyslexia. Dyslexia.

[66] Weiss A., Granot R., Ahissar M. (2014). The enigma of dyslexic musicians. Neuropsychologia.

[67] Gordon R.L., Fehd H.M., McCandliss B.D. (2015). Does Music Training Enhance Literacy Skills? A Meta-Analysis. Front. Psychol..

[68] Abrams D.A., Nicol T., Zecker S., Kraus N. (2009). Abnormal Cortical Processing of the Syllable Rate of Speech in Poor Readers. J. Neurosci..

[69] Leong V., Goswami U. (2014). Assessment of rhythmic entrainment at multiple timescales in dyslexia: Evidence for disruption to syllable timing. Hear. Res..

[70] Rosen S. (1992). Temporal Information in Speech: Acoustic, Auditory and Linguistic Aspects. Philos. Trans. R. Soc. B Biol. Sci..

[71] Kraus N., Slater J., Thompson E.C., Hornickel J., Strait D.L., Nicol T., White-Schwoch T. (2014). Music Enrichment Programs Improve the Neural Encoding of Speech in At-Risk Children. J. Neurosci..

[72] Tichko P., Skoe E. (2017). Frequency-dependent fine structure in the frequency-following response: The byproduct of multiple generators. Hear. Res..

[73] Coffey E.B.J., Herholz S.C., Chepesiuk A.M.P., Baillet S., Zatorre R.J. (2016). Cortical contributions to the auditory frequency-following response revealed by MEG. Nat. Commun..

[74] Bidelman G.M. (2015). Multichannel recordings of the human brainstem frequency-following response: Scalp topography, source generators, and distinctions from the transient ABR. Hear. Res..

[75] Bidelman G.M. (2018). Subcortical sources dominate the neuroelectric auditory frequency-following response to speech. Neuroimage.

[76] Hall J. (1992). Handbook of Auditory Evoked Responses.

[77] Eggermont J.J., Don M. (1986). Mechanisms of Central Conduction Time Prolongation in Brain-Stem Auditory Evoked Potentials. Arch. Neurol..

[78] Poeppel D., Idsardi W.J., van Wassenhove V., Wassenhove V. (2008). Van Speech perception at the interface of neurobiology and linguistics. Philos. Trans. R. Soc. B Biol. Sci..

[79] Skoe E., Tufts J. (2018). Evidence of noise-induced subclinical hearing loss using auditory brainstem responses and objective measures of noise exposure in humans. Hear. Res..

[80] Liberman M.C., Epstein M.J., Cleveland S.S., Wang H., Maison S.F. (2016). Toward a differential diagnosis of hidden hearing loss in humans. PLoS ONE.

[81] Reiman M., Parkkola R., Johansson R., Jääskeläinen S.K., Kujari H., Lehtonen L., Haataja L., Lapinleimu H. (2009). Diffusion tensor imaging of the inferior colliculus and brainstem auditory-evoked potentials in preterm infants. Pediatr. Radiol..

[82] Otto-Meyer S., Krizman J., White-Schwoch T., Kraus N. (2018). Children with autism spectrum disorder have unstable neural responses to sound. Exp. Brain Res..

[83] Corrigall K., Trainor L. (2011). Associations between length of music training and reading skills in children. Music Percept..

[84] Slater J., Kraus N. (2016). The role of rhythm in perceiving speech in noise: A comparison of percussionists, vocalists and non-musicians. Cogn. Process..

[85] Skoe E., Krizman J., Anderson S., Kraus N. (2015). Stability and plasticity of auditory brainstem function across the lifespan. Cereb. Cortex.

[86] Chiappa K., Gladstone K., Young R. (1979). Brain stem auditory evoked responses: Studies of waveform variations in 50 normal human subjects. Arch. Neurol..

[87] Don M., Allen A.R., Starr A. (1977). Effect of Click Rate on the Latency of Auditory Brain Stem Responses in Humans. Ann. Otol. Rhinol. Laryngol..

[88] Benjamini Y., Hochberg Y. (1995). Controlling the false discovery rate: A practical and powerful approach to multiple testing. J. R. Stat. Soc. B.

[89] Moore E., Schaefer R., Bastin M., Roberts N., Overy K. (2014). Can Musical Training Influence Brain Connectivity? Evidence from Diffusion Tensor MRI. Brain Sci..

[90] Kim S.G., Knösche T.R. (2016). Intracortical myelination in musicians with absolute pitch: Quantitative morphometry using 7-T MRI. Hum. Brain Mapp..

[91] Imfeld A., Oechslin M.S., Meyer M., Loenneker T., Jancke L. (2009). White matter plasticity in the corticospinal tract of musicians: A diffusion tensor imaging study. Neuroimage.

[92] Giacosa C., Karpati F.J., Foster N.E.V., Penhune V.B., Hyde K.L. (2016). Dance and music training have different effects on white matter diffusivity in sensorimotor pathways. Neuroimage.

[93] Boebinger D., Evans S., Rosen S., Lima C.F., Manly T., Scott S.K., Lima C.F., Manly T., Scott S.K. (2015). Musicians and non-musicians are equally adept at perceiving masked speech. J. Acoust. Soc. Am..

[94] Slater J., Skoe E., Strait D.L., O’Connell S., Thompson E., Kraus N., O’Connell S., Thompson E., Kraus N. (2015). Music training improves speech-in-noise perception: Longitudinal evidence from a community-based music program. Behav. Brain Res..

[95] Ruggles D.R., Freyman R.L., Oxenham A.J. (2014). Influence of musical training on understanding voiced and whispered speech in noise. PLoS ONE.

[96] Goswami U. (2015). Sensory theories of developmental dyslexia: Three challenges for research. Nat. Rev. Neurosci..

[97] Chan A.S., Ho Y.C., Cheung M.C. (1998). Music training improves verbal memory. Nature.

[98] Hanna-pladdy B., Mackay A. (2011). The relation between instrumental musical activity and cognitive aging. Neuropsychology.

[99] Ho Y.-C., Cheung M.-C., Chan A.S. (2003). Music training improves verbal but not visual memory: Cross-sectional and longitudinal explorations in children. Neuropsychology.

[100] Parbery-Clark A., Skoe E., Kraus N. (2009). Musical experience limits the degradative effects of background noise on the neural processing of sound. J. Neurosci..

[101] Bigand E., Delbé C., Poulin-Charronnat B., Leman M., Tillmann B. (2014). Empirical evidence for musical syntax processing? Computer simulations reveal the contribution of auditory short-term memory. Front. Syst. Neurosci..

[102] Kraus N., Strait D.L., Parbery-Clark A. (2012). Cognitive factors shape brain networks for auditory skills: Spotlight on auditory working memory. Ann. N. Y. Acad. Sci..

[103] Parbery-Clark A., Skoe E., Lam C., Kraus N. (2009). Musician enhancement for speech-in-noise. Ear Hear..

[104] Law L.N.C., Zentner M. (2012). Assessing Musical Abilities Objectively: Construction and Validation of the Profile of Music Perception Skills. PLoS ONE.

[105] Skoe E., Krizman J., Kraus N. (2013). The impoverished brain: Disparities in maternal education affect the neural response to sound. J. Neurosci..

[106] Hille K., Gust K., Bitz U., Kammer T. (2011). Associations between music education, intelligence, and spelling ability in elementary school. Adv. Cogn. Psychol..

